# Sequence Analysis of the IL28A/IL28B Inverted Gene Duplication That Contains Polymorphisms Associated with Treatment Response in Hepatitis C Patients

**DOI:** 10.1371/journal.pone.0029983

**Published:** 2012-01-10

**Authors:** Jennifer M. Reynolds, Sara A. Paciga, Frances A. Sanders, Craig L. Hyde, A. Katrina Loomis, Geoffrey I. Johnston

**Affiliations:** 1 DNA and BioFluids Research Center of Emphasis, Pfizer Global Research and Development, Groton, Connecticut, United States of America; 2 Precision Medicine, Pfizer Global Research and Development, Groton, Connecticut, United States of America; 3 Precision Medicine, Pfizer Global Research and Development, Sandwich, Kent, United Kingdom; 4 Biostatistics, Pfizer Global Research and Development, Groton, Connecticut, United States of America; Duke University School of Medicine, United States of America

## Abstract

Several SNPs located in or around the *IL28B* gene are associated with response of patients infected with Hepatitis C virus to treatment with pegylated interferon-α +/− ribavirin or with spontaneous clearance of the virus. The results of such studies are so compelling that future treatment approaches are likely to involve clinical decisions being made on the basis of a patient's genotype. Since *IL28B* is a paralogue of *IL28A* with greater than 95% sequence identity, it is possible that without genotyping assay specificity, sequences in *IL28A* may contribute to genotype identification, and potentially confound treatment decisions. This study aimed to 1) examine DNA sequences in *IL28B* surrounding each of the reported associated SNPs and the corresponding regions in *IL28A*; and 2) develop a robust assay for rs12979860, the most ‘cosmopolitan’ SNP most strongly associated with treatment response across all global populations studied to date. Bioinformatic analysis of genomic regions surrounding *IL28A* and *IL28B* demonstrated that 3 SNPs were unique to *IL28B*, whereas the remaining 6 SNP regions shared >93% identity between *IL28A* and *IL28B*. Using a panel of DNA samples, PCR amplification followed by Sanger sequencing was used to examine *IL28B* SNPs and the corresponding regions in *IL28A*. For the overlapping SNPs, all 6 in *IL28B* were confirmed to be polymorphic whereas the corresponding positions in *IL28A* were monomorphic. Based upon *IL28A* and *IL28B* sequence data, a specific TaqMan® assay was developed for SNP rs12979860 that was 100% concordant to the sequence-derived genotypes. Analysis using a commercial assay identified one discordant result which led to a change in their genotype-calling algorithm. Where future treatment decisions are made upon the results of genotyping assays, it is very important that results are concordant with data from a sequence-based format. This is especially so in situations where designing specific PCR primers is a challenge.

## Introduction

Worldwide there are over 170 million individuals who are chronically infected with Hepatitis C virus (HCV) [1]. Frontline therapy is currently pegylated interferon-α either alone or in combination with ribavirin, although new direct-acting antiviral treatments (boceprevir and telaprevir) [2], [3] have recently received FDA approval. It was known that sustained virological response (SVR) rates following interferon treatment varied amongst individuals and across different populations suggesting that a genetic component contributed to treatment response. In late 2009 genetic evidence supporting this was identified through the use of genome-wide association studies. Several single nucleotide polymorphisms (SNPs) located in and around the *IL28B* gene were found to be associated with treatment response in patients chronically infected with HCV [4]–[6]. In total, these papers identified 9 SNPs that were either associated with increased SVR or with a null virological response (NVR). In addition, some of these SNPs were also associated with spontaneous clearance of HCV [7]. Since these original studies, there have now been hundreds of papers examining genotype associations with some of these 9 SNPs. In addition, HCV treatment algorithms that include patient genotype are now being considered [8], [9].

*IL28B* lies in close proximity on chromosome 19 to its paralogue *IL28A*, a gene with which it shares >95% sequence identity [10]. This high level of sequence identity between the 2 genes raised a potential concern that sequences within *IL28A* may interfere with genotype determination unless a robust, validated genotyping assay was developed. To address this potential concern, this study sought to carry out a detailed bioinformatic analysis of the regions surrounding the *IL28A* and *IL28B* gene duplication and to genotype a number of samples by sequencing the DNA surrounding the SNPs in *IL28B* as well as the corresponding region of *IL28A*. Finally, a specific and robust genotyping assay was developed for rs12979860, the SNP most strongly associated with treatment response across all global populations studied to date [8].

## Methods

### Bioinformatic analysis

A 43 kb fragment of chromosome 19 encompassing the sequences of *IL28A* and *IL28B* (corresponding to nucleotides 39,727,000–39,770,000 from NCBI Genome Build 37) was extracted and subjected to bioinformatic analysis using Clustal W [11]. Since *IL28A* and *IL28B* are found on the forward and reverse strands, respectively, an alignment comparing the extracted sequence to the reverse complement of the same sequence was performed. The locations of each of the SNPs rs8105790, rs11881222, rs8103142, rs28416813, rs4803219, rs12979860, rs12980275, rs8099917 and rs7248668 were identified on the resulting alignment.

### Sequence analysis

A panel of 48 genomic DNA samples was obtained from the Coriell Institute (Camden, NJ). These comprised 16 samples each from individuals of Caucasian (CEU), Japanese (JPT) and Yoruba (YRI) origins. PCR amplification of 8 regions spanning the 9 SNP locations and the corresponding regions of *IL28A* was performed using the HotStarTaq PCR kit (Qiagen, Germantown, MD). The PCR reactions (25 uL) included 25 ng of DNA and amplifying primers at a final concentration of 0.4 µM. After enzyme activation at 95°C for 15 minutes, there were 35 PCR cycles comprising 1 minute denaturation at 94°C, 1 minute at the appropriate annealing temperature and 3 minutes extension at 72°C. The last cycle was followed by a final extension at 72°C for 10 minutes. Some amplicons required the use of Q-Solution (Qiagen) as 20% of the reaction volume for amplification. Fragment sizes ranged from 476–2500 bp (Table 1).

**Table 1:** Details of PCR amplicons used to examine SNP locations in *IL28B* and the corresponding reverse complement (RC) regions of *IL28A*.

Target Gene	Fragment Size (bp)	SNPs covered	Amplifying Primer Sequences	Annealing Temp. (°C)	20% Q Solution
*IL28B*	1697	rs12980275	5′ AGCAAGAGGAGGGAAGGAAG 3′	64	No
		rs8105790	5′ CCCTGGATAAGCCCCTACAG 3′		
*IL28B*	1115	rs11881222	5′ TGGGTGTCTTTTCCTCATTG 3′	58	No
		rs8103142	5′ CCTCCAATCCCATCAGAG 3′		
*IL28B*	919	rs28416813	5′ TGACCCTTGGAGTGCGGG 3′	60	Yes
		rs4803219	5′ ATATGCCAGGAGTGGTGG 3′		
*IL28B*	593	rs12979860	5′ CCAGCAGCTCCAGGATCG 3′	58	Yes
			5′ GCAGGCGCCTCTCCTATG 3′		
*IL28B*	1291	rs8099917	5′ TCACCATCCTCCTCTCATCC 3′	64	No
		rs7248668	5′ GCACCCAAAGCCTAACCATA 3′		
*IL28A*	476	RC of rs12979860	5′ ACACACCCGTCGCTGACC 3′	60	Yes
			5′ CTCTCCCGTCCGCTTCTG 3′		
*IL28A*	2500	RC of rs11881222	5′ GCCAATTGGTGAACTGTCAT 3′	60	No
		RC of rs8103142	5′ CCCAGCTCATCAAGTGTGTCT 3′		
		RC of rs28416813			
		RC of rs4803219			
*IL28A*	990	RC of rs8105790	5′ CCACAAATGAGGGGGACA 3′	60	No
			5′ TGGCCAGCTGGTTCTTCTAC 3′		

Primers were designed for specificity to either *IL28A* or *IL28B* by choosing primer annealing sites with the greatest number of mismatches between the two genes. To further avoid mispriming from homologous regions, the annealing temperature was determined empirically for each set of primers using a gradient cycle from 50°C to 68°C on test samples prior to amplifying from the 48 DNA sample panel. In all cases, the highest annealing temperature that produced a single robust band on a gel and specific *IL28A* or *IL28B* sequence data was used for subsequent amplification from the complete panel (Table 1).

Five PCR fragments spanned regions in the *IL28B* gene and 3 fragments covered the corresponding regions of *IL28A*. Purification and direct Sanger sequencing of the products was performed by Beckman Coulter Genomics (Morrisville, NC). All products were sequenced either using their amplification primers or using additional internal sequencing primers (see Table S1). The resulting data was assembled and analysed using Sequencher 4.7 (Gene Codes Corporation, Ann Arbor, MI). Representative sequence traces for each of the SNP locations and genotypes are given in the Figure S1.

### TaqMan® genotyping assay

A specific TaqMan® genotyping assay was developed for rs12979860 using Qiagen's Type- it® Fast SNP Probe PCR chemistry. Primer and probe oligonucleotides were designed following as closely as possible to the default parameters of Primer Express 3.0 (Applied Biosystems Inc., Foster City, CA), however, in order to choose primers that annealed to specific locations in *IL28B*, some exceptions were made (such as product size, nucleotide runs and Tm). Primers amplifying a 214 bp product that was specific to *IL28B* (Table 2) were manufactured by Applied Biosystems Inc. Thermal cycling was performed on the ABI 9700 thermal cycler (Applied Biosystems Inc.) using 10 ng DNA as the template. Enzyme activation and the initial denaturation cycle was at 95°C for 5 minutes, followed by 40 cycles of 95°C for 15 seconds and 60°C for 1 minute. Plates were read on an ABI 7900HT (Applied Biosystems Inc.) and the data was analyzed using SDS 2.3 software (Applied Biosystems Inc.).

**Table 2:** TaqMan® amplification primers and detection probes for the rs12979860 genotyping assay.

Name	Detector	Sequence 5′ - 3′
Forward Primer		TTGCGC**TG**CC**C**CCA**G**
Reverse Primer		GGAG**C**GCGG**AG**TGCAA
Probe1	VIC	TCCCCGAAGGCGTGA
Probe2	FAM	CGAAGGCGCGAAC

Nucleotides that gave the amplifying primers their specificity for *IL28B* are shown in bold underlined. The SNP location in the probes is indicated by the underlined nucleotide in larger font.

### Comparison to a commercial rs12979860 assay

A subset of 24 of the 48 genomic DNA samples that had been sequenced and genotyped were selected for rs12979860 SNP analysis by Monogram Bioscience Inc. (San Francisco, CA). Nine samples were selected to represent the CC genotype, 8 samples to represent CT genotype and 7 samples to represent TT genotype. Aliquot parts of the same DNA samples that had been sequenced and genotyped were submitted for analysis. No details of the testing procedure or analytical validity of the LabCorp Inc. assay have been published [12].

## Results

### Bioinformatics

**Figure 1:**
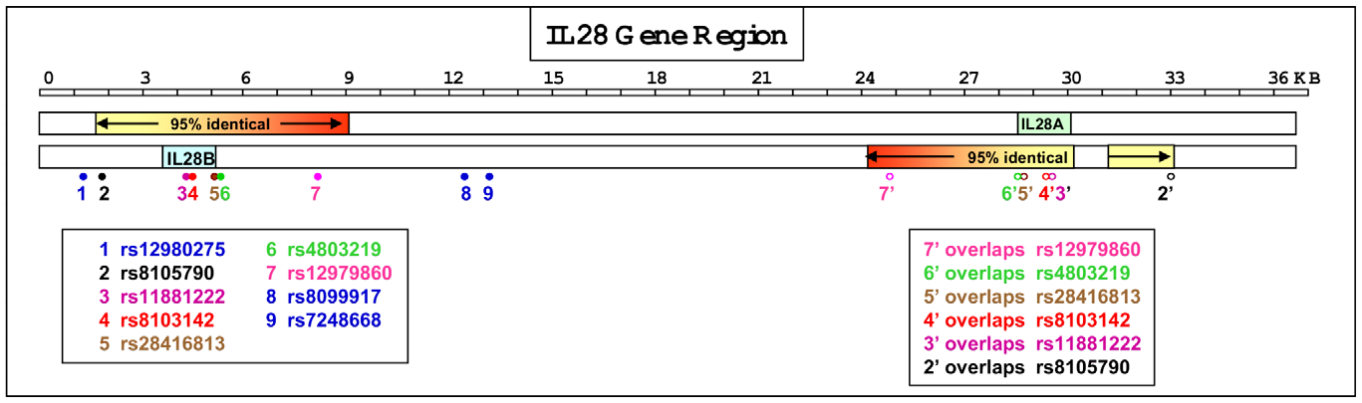
Schematic showing IL28 genes, SNP locations and areas of sequence homology. Illustration of the relative locations of the *IL28A* and *IL28B* genes on the forward and reverse DNA strands on chromosome 19. The shaded boxes indicate regions of the inverted duplication with >95% sequence identity. The direction of the shaded gradient indicates the orientation of the match. SNP identifiers in blue were unique to the *IL28B* region, whereas SNPs highlighted in other colors were present in the *IL28B* region as well as a corresponding region in *IL28A*.

**Figure 2:**
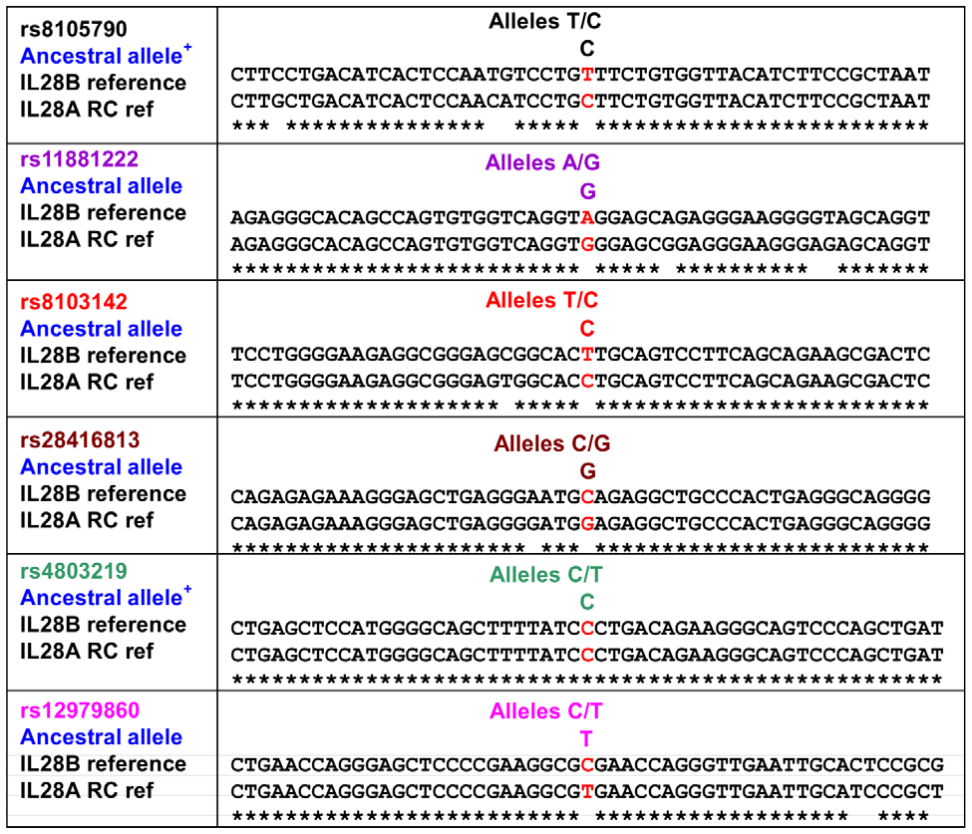
Sequence alignments surrounding six of the treatment response associated SNPs. Alignment comparing 50 bp sequence surrounding SNP locations in *IL28B* reference sequence from NCBI genome build 37 and the corresponding reverse complement regions of *IL28A* reference sequence (RC ref). SNP identifiers (color coded to match Figure 1) and their respective alleles are shown. Ancestral alleles were obtained from dbSNP, except for those indicated by +; in these cases, the allele was based upon alignment of gorilla, chimpanzee, macaque and orangutan sequences. SNP locations on each alignment are highlighted in red. The asterisk denotes sequence identity.

Analysis of the IL28 gene region confirmed 97.5% sequence identity between the coding exons and introns of the *IL28A* and *IL28B* genes. In addition, the analysis also showed that a 7.3 kb region extending both upstream and downstream of the two genes shared greater than 95% sequence identity (illustrated in Figure 1). Six of the 9 reported SNPs associated with pegylated interferon treatment response in HCV patients were located within this 7.3 kb block and each of these SNPs matched regions present in the DNA sequence surrounding *IL28A* (Figure 2). Interestingly, one of the 2 alleles across each 6 SNP locations in the *IL28B* reference sequence (NCBI Genome Build 37) exactly matched the corresponding position in *IL28A* and the ancestral allele. For example, for rs12979860 the two alleles reported for this SNP were C and T. The reference nucleotide at this location in *IL28B* was C, whereas it was a T in the reverse complement of IL28A and the ancestral allele was also a T. This finding, together with the high levels of sequence identity across the 7.3 kb region, raised theoretical questions about the potential specificity of genotyping assays for 6 of the 9 reported SNPs. To address this matter, a PCR amplification and Sanger sequencing approach was taken to obtain definitive DNA sequence data to confirm the presence of SNPs in *IL28B* and to enable assay development for the most cosmopolitan SNP, rs12979860.

### Sequence analysis

For each of 48 different DNA samples, 8 regions spanning SNPs within and surrounding *IL28B*, as well as the corresponding regions of *IL28A*, were amplified by PCR and the resulting products were sequenced directly using BigDye® terminator chemistry (Applied Biosystems Inc.). Sequences were assembled to provide a consensus sequence for each PCR fragment and the alleles at each SNP location identified. All 9 SNPs previously identified that were associated with pegylated interferon treatment response were found to be polymorphic, whereas the 6 overlapping regions in *IL28A* were all monomorphic (Tables 3 and 4). Allele frequencies for the CEU, JPT and YRI populations were similar to those reported in dbSNP.

**Table 3:** Allele frequencies of SNPs unique to *IL28B* region.

SNP	rs12980275	rs8099917	rs7248668
**Alleles**	A/G	T/G	G/A
Freq - CEU	0.56/0.44	0.75/0.25	0.75/0.25
Freq - JPT	0.91/0.09	0.91/0.09	0.91/0.09
Freq - YRI	0.47/0.53	0.97/0.03	0.97/0.03

**Table 4:** Allele frequencies of *IL28B* SNPs and corresponding regions of *IL28A*.

	SNP in *IL28B* region					
SNP	rs8105790	rs11881222	rs8103142	rs28416813	rs4803219	rs12979860
**Alleles**	T/C	A/G	T/C	C/G	C/T	C/T
Freq - CEU	0.75/0.25	0.59/0.41	0.56/0.44	0.63/0.37	0.66/0.34	0.53/0.47
Freq - JPT	0.91/0.09	0.91/0.09	0.91/0.09	0.91/0.09	0.91/0.09	0.91/0.09
Freq - YRI	0.78/0.22	0.69/0.31	0.31/0.69	0.34/0.66	0.66/0.34	0.34/0.66

	Corresponding base in *IL28A* (reverse complement) sequence overlapping *IL28B*					
	rs8105790	rs11881222	rs8103142	rs28416813	rs4803219	rs12979860
**Alleles**	C	G	C	G	C	T
Freq - CEU	1.00	1.00	1.00	1.00	1.00	1.00
Freq - JPT	1.00	1.00	1.00	1.00	1.00	1.00
Freq - YRI	1.00	1.00	1.00	1.00	1.00	1.00

By carrying out a sequence-based analysis of PCR fragments spanning the *IL28A* and *IL28B* loci, a total of 17 novel SNPs were also identified across the 48 samples; one was present in *IL28A* and the remaining 16 were in *IL28B*. Of these 17 SNPs, all were present in non-coding regions except for one, E95Q, that was present in an *IL28B* protein-coding exon. The novel SNPs were all confirmed in double stranded sequence. Each SNP was a heterozygote and they were only present in one or two individual samples (Table S2).

### Assay for rs12979860

**Figure 3:**
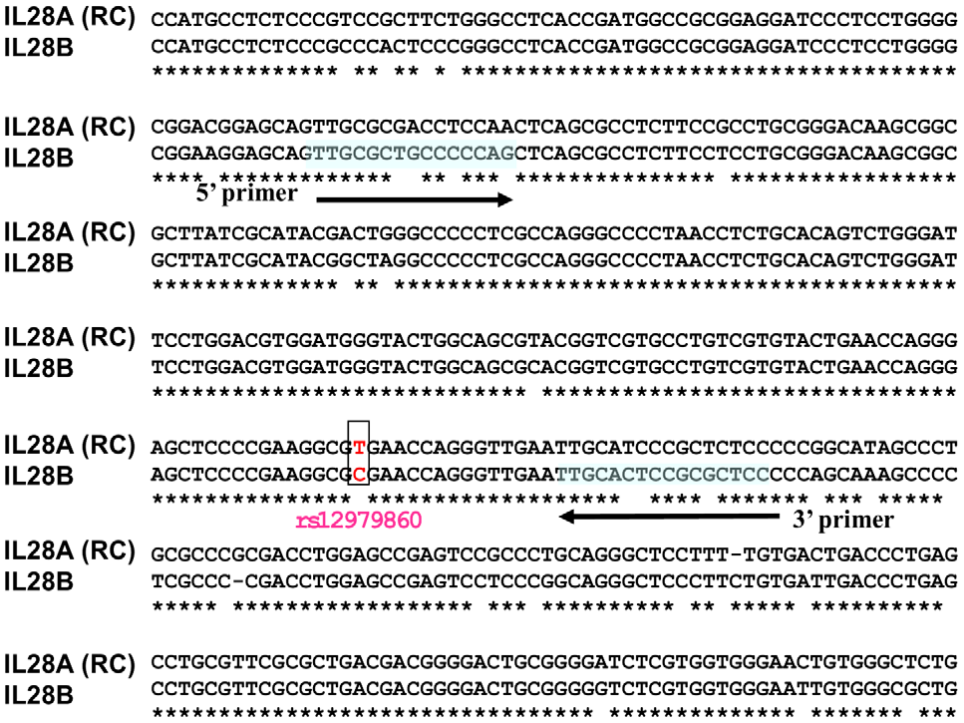
Sequence alignment of *IL28B* and *IL28A* used to design the rs12979860 genotyping assay. Clustal W alignment of a portion of the *IL28B* gene surrounding rs12979860 to the reverse complement (RC) of the corresponding region in *IL28A*. Locations of the genotyping assay primers are shaded and the rs12979860 SNP is boxed and highlighted in red.

**Figure 4:**
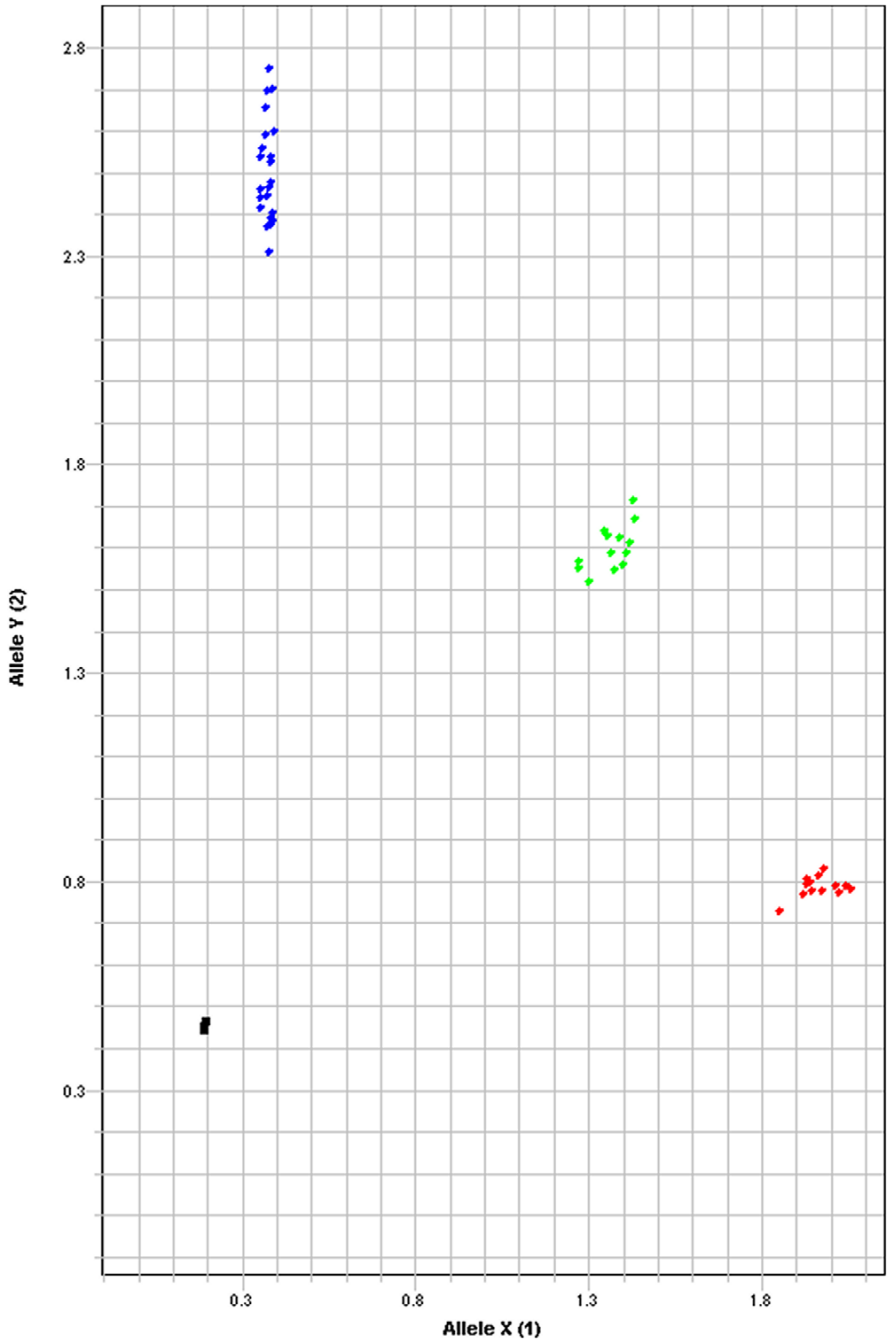
Allelic discrimination plot for the rs12979860 genotyping assay. Allelic discrimination plot generated by the SDS 2.3 software (Applied Biosystems Inc.) for the rs12979860 genotyping assay using the 48 sample DNA panel. Red and blue circles represent the two homozygous genotypes (T/T and C/C), the green circles represent heterozygous genotype (C/T) and the black squares represent no template control.

rs12979860 has become the most widely studied SNP in the HCV field since the original publications reporting the results of genome-wide association studies. Using the consensus DNA sequence of the fragment containing this SNP, a sensitive TaqMan® based assay was developed. The specificity of the assay was driven by the amplification primers (Figure 3). Specifically, the forward primer had 4 base mismatches compared to *IL28A*, including the final base at the terminal 3′ end, and the reverse primer had 3 base mismatches. Although the VIC-labeled probe sequence was common to both *IL28A* and *IL28B*, the specific amplification of *IL28B* allowed for gene-specific discrimination as illustrated in Figure 4.

The TaqMan® assay gave results that were 100% concordant with the sequence based data using the 48 DNA samples previously examined. To determine the lower confidence limits of the concordance, a Beta distribution was used. The distribution of underlying true probabilities generating ‘s’ successes in ‘n’ trials is given by Beta(p, s+1, n–s+1), and this was used to determine that the underlying 99% and 95% lower confidence limits on concordance were 91.03% and 94.07%, respectively.

The genotype results of all 48 Coriell Institute DNA samples are listed in the Table S3), alongside sequence traces used to make the rs12979860 genotype calls (Figure S2).

### Comparison with other data sources

HapMap data for rs12979860 was available for the JPT and YRI samples (Table S3). Genotypes for 30 of 32 samples were 100% concordant, with no data available for two samples. In addition, a subset of 24 out of the 48 DNA samples were also analyzed by Monogram Biosciences Inc. using a commercial assay developed by LabCorp Inc. The results were concordant for 23 out of 24 samples analyzed (Table S3). The discordant sample (Coriell identifier NA18502) was a C/T heterozygote in both the Sanger sequencing and TaqMan assays, but called a T/T homozygote in the LabCorp assay. This sample was one of the two HapMap samples with missing data.

To confirm that there had been no sample mix-up with NA18502 in the author's lab, a total of 8 SNPs from 4 chromosomes were determined in addition to a gender marker. All 9 markers were fully concordant with HapMap data suggesting that there was <1% chance of a sample mix up (Table S4).

Upon further analysis at Monogram Biosciences Inc., the T/T signals obtained for sample NA18502 were found to be inconsistent with the normal distribution of T/T results observed in the assay. The interpretation algorithm now used at Monogram Biosciences Inc. has been modified to account for this variant result.

The Coriell Institute DNA samples are publically available, therefore the same samples could be used in the future to benchmark newly developed assays.

## Discussion

Genome-wide association studies have demonstrated that SNPs found in the region surrounding *IL28B* were associated with response of patients infected with HCV to treatment with pegylated interferon-α (with or without ribavirin) [4]–[6] and with spontaneous clearance of HCV infection [7]. Over the last two years, the number of publications examining *IL28B* SNPs has increased tremendously and treatment options based upon a patient's *IL28B* SNP genotype are being considered [8], [9].

The *IL28B* gene has a close paralogue, *IL28A*, with which it shares 97.5% sequence identity. This raised the possibility that sequences in *IL28A* may contribute to the determination of *IL28B* SNP genotypes. Therefore, understanding genotype assay specificity would be vitally important to consider when using *IL28B* genotypes to make treatment decisions. Clinicians need to be confident that ‘homebrew’ assays are robust and fully validated to allow prospective genotyping to make treatment decisions on single subjects rather than assays for retrospective cohort-based analyses.

The *IL28A* and *IL28B* genes likely arose due to an ancestral gene duplication and inversion event, with *IL28A* being located on the positive DNA strand of chromosome 19 and *IL28B* 24 kb downstream on the negative strand. Although these genes are 97.5% identical across their 1.4 kb length, a region of >95% sequence identity stretches a total of 7.3 kb in both 5′ and 3′ directions and it includes 6 of the 9 reported treatment response associated SNPs. Our observation that in all cases, one of the two alleles of the *IL28B* SNPs also corresponded to identical nucleotides in *IL28A* led us to take a sequence-based approach in order to confirm SNP genotypes. Sequencing specific regions from a panel of DNA samples from 48 individuals representing CEU, JPT and YRI origins demonstrated that all 9 SNPs in the *IL28B* region were polymorphic, whereas the corresponding sequences in *IL28A* were all monomorphic. Despite this finding, there was still the potential for *IL28A* sequences to contribute to genotyping calls if assays were not sufficiently specific.

As a general rule when developing PCR-based genotyping assays, the greater the number of mismatches between the target primers and other homologous sequences, the greater the assay specificity. SNP rs12979860 currently appears to be the best single choice variant for diagnostic purposes across global populations or for use in clinical trials [8] and as a result, it has now become the most studied SNP of the 9 treatment response associated SNPs that were initially identified. Our TaqMan® assay for rs12979860 used two PCR primers that contained 3 and 4 base mismatches per primer compared to the corresponding *IL28A* sequences. This assay gave 100% concordant results to the sequence-derived genotypes. Whilst not implying that published rs12979860 assays have not been technically validated, examining a selection of different types of rs12979860 genotyping assays found varying numbers of base mismatches in the PCR amplification primers. For example, one TaqMan® assay had 0 and 3 mismatches in the amplification primer pairs [13], [14], a PCR/Sybr green assay had 0 and 2 mismatches [15], and a MELT-MAMA PCR assay had 0 and 5 mismatches [16]. In these three examples, the specificity for *IL28B* is only driven by a single primer. Two commercially available rs12979860 assays from LabCorp Inc and Quest Diagnostics Inc. have not reported details of their respective assay components. Analysis of a 24 sample subset of the 48 Coriell DNAs revealed one discordant genotype using the LabCorp assay; interestingly in a sample that had its genotype missing in the HapMap data. Re-analysis of this discordant data-point led to a modification of the genotype calling algorithm now used at Monogram Biosciences Inc.

Given the importance of the studies being used worldwide to examine associations between SNP rs12979860 and response to treatment in various hepatitis populations, it is vitally important that robust and well validated assays are used. This is particularly important as future hepatitis patient treatment decisions will likely be dependent upon results obtained from such genotyping assays.

## Supporting Information

Figure S1 **Representative sequencing chromatograms covering the 9** ***IL28B*** **SNPs as well as the corresponding regions in** ***IL28A*** **.** Sanger sequencing chromatograms illustrating the different genotypes found in different Coriell samples for each *IL28B* SNP and the corresponding regions in *IL28A*.
(DOC)

Figure S2 **Sequencing chromatograms covering the** ***IL28B*** **rs12979860 SNP for the 48 Coriell Institute DNA sample panel.** Sanger sequence chromatograms for the CEU sample panel (NA17233 - NA17297), the JPT sample panel (NA18954 -NA19085) and the YRI sample panel (NA18502 - NA19223).
(DOC)

Table S1 **Primers used to sequence PCR amplicons.** Details of sequencing primers used to examine the PCR amplicons encompassing SNP locations in *IL28B* and the corresponding reverse complement (RC) regions of *IL28A*.
(DOC)

Table S2 **Novel SNPs identified in** ***IL28A*** **and** ***IL28B*** **gene regions in DNA samples from the Coriell Institute.** SNP location and corresponding alleles are indicated using the IUPAC ambiguity codes (R, S, W, Y).
(DOCX)

Table S3 **Genotypes determined for Coriell Institute DNA samples using DNA sequencing, TaqMan® and LabCorp genotyping assays and data extraction from HapMap.** Sample NA18502 (bold) is the sample with the discordant result in the LabCorp Inc. assay. n/n indicates the 2 samples that were genotyped in HapMap project, but no results were reported.
(DOCX)

Table S4 **Concordance of additional genotypes determined for Coriell Institute DNA sample NA18502 compared to HapMap data (Release 24).** r^2^ values from linkage disequilibrium analysis in the YRI population were 0.12 for rs2032586 and rs2235015 on chromosome 6; on chromosome 7 they were 0.06 for rs622342 and rs316019; and on chromosome 19 they were 0.48 for rs8105790 and rs11881222; 0.08 for rs8105790 and rs7248668; and 0.05 for rs11881222 and rs7248668. Analysis also determined that NA18502 was from a female.
(DOCX)
