# Supplementary material for: Sequence Analysis of the IL28A/IL28B Inverted Gene Duplication That Contains Polymorphisms Associated with Treatment Response in Hepatitis C Patients
Source: PLoS One. 2012 Jan 10;7(1):e29983. doi: 10.1371/journal.pone.0029983 (PMC3254624; doi:10.1371/journal.pone.0029983)

**Figure S1 Representative sequencing chromatograms covering the 9 *IL28B* SNPs as well as the corresponding regions in *IL28A***

***IL28B* – rs12980275**

A/A (Sample NA17238)


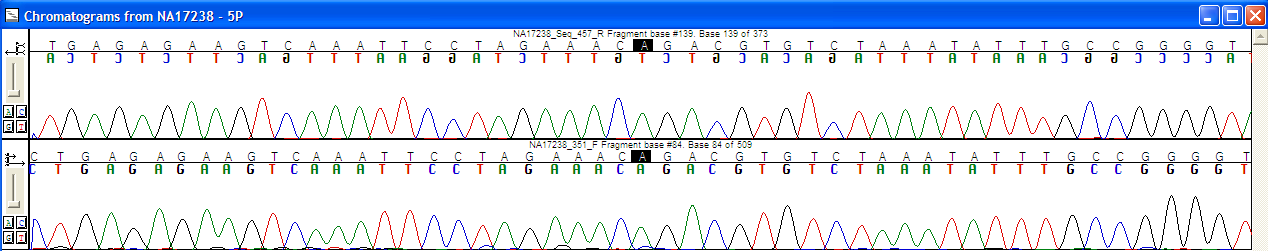


A/G (Sample NA17233)


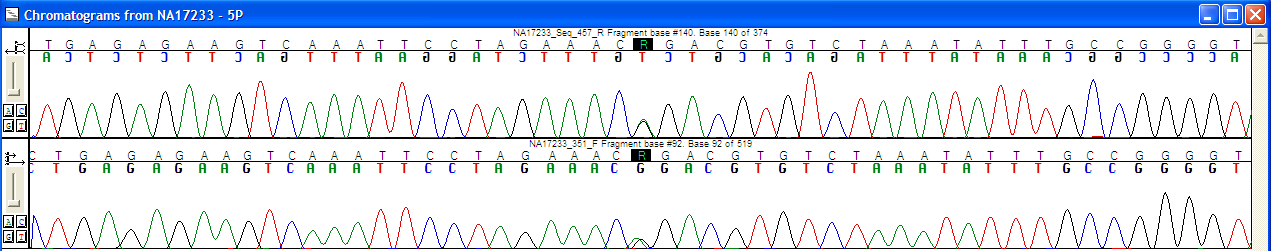


G/G (Sample NA17234)


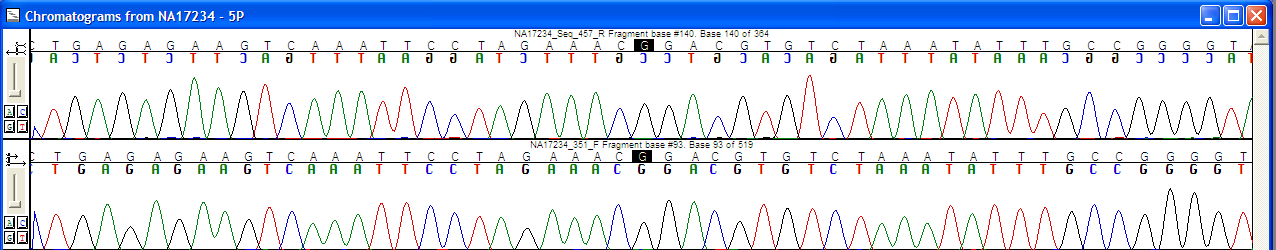


***IL28B* – rs8105790**

T/T (Sample NA18502)


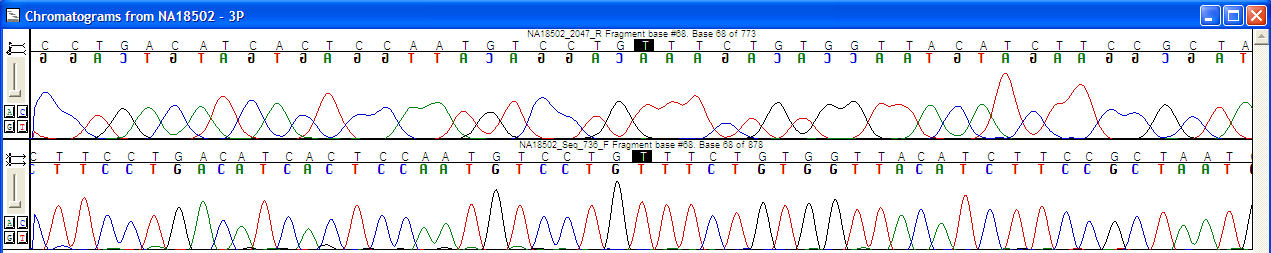


T/C (Sample NA18955)


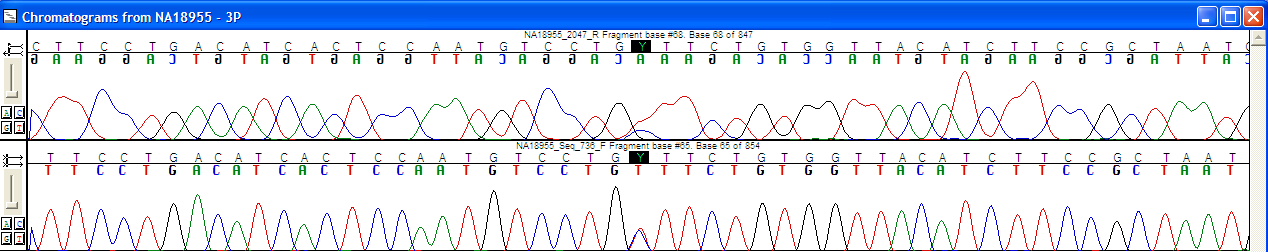


***IL28B* – rs11881222**

A/A (Sample NA18858)


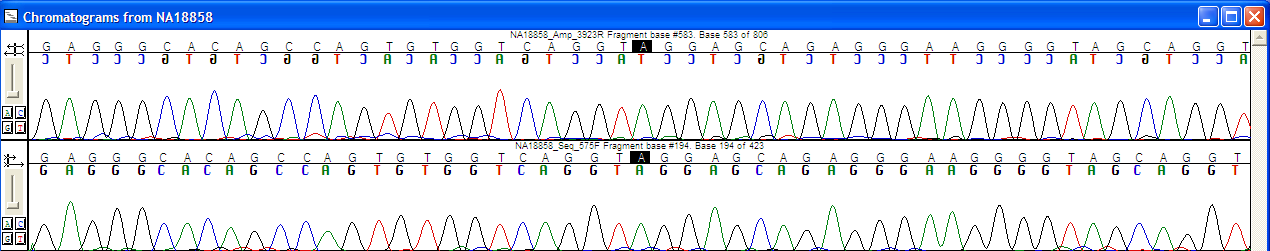


A/G (Sample NA18506)


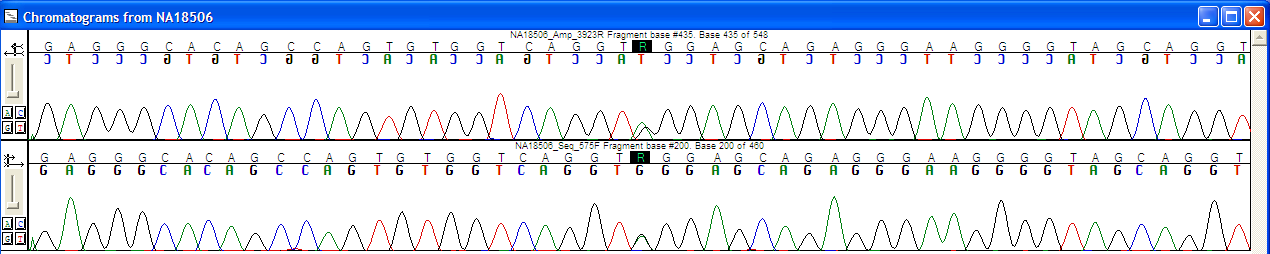


G/G (Sample NA18504)


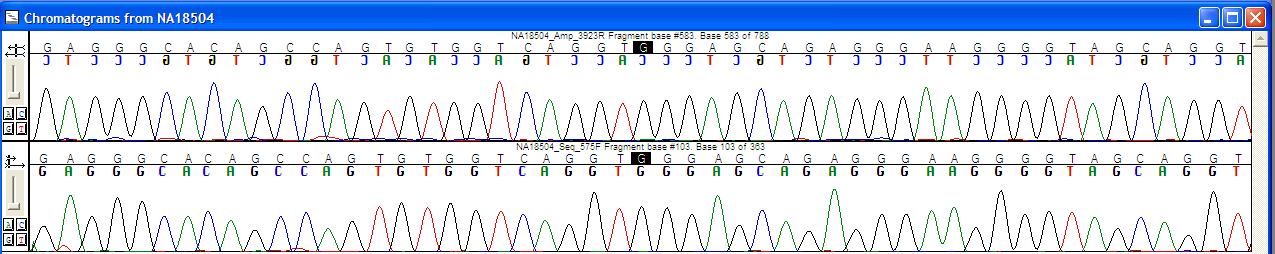


***IL28B* – rs8103142**

T/T (Sample NA17243)


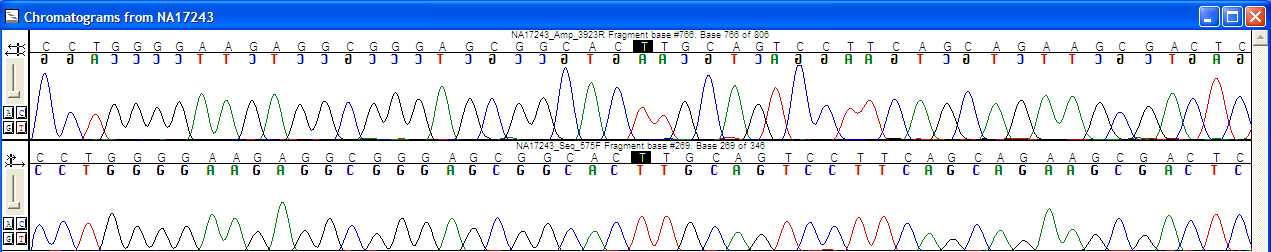


T/C (Sample NA17235)


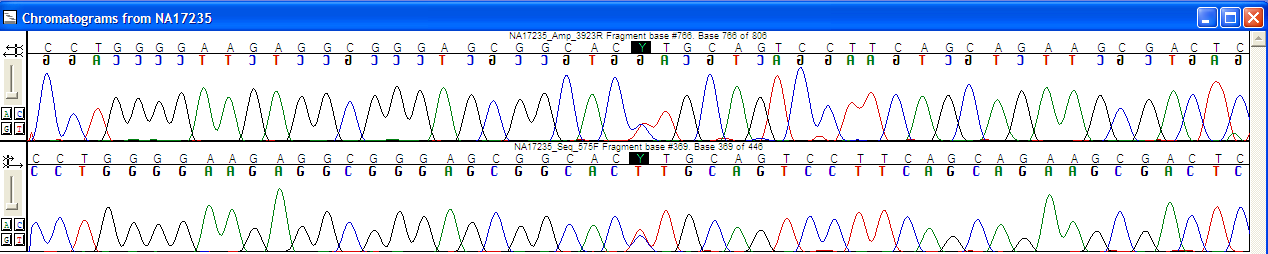


C/C (Sample NA17241)


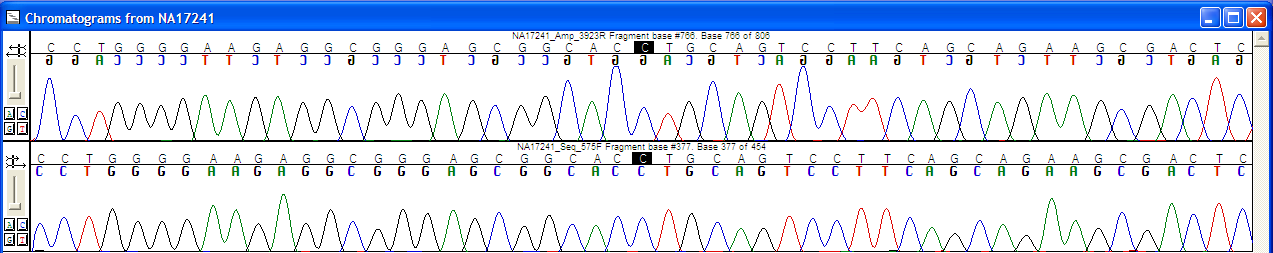


***IL28B* – rs28416813**

C/C (Sample NA19002)


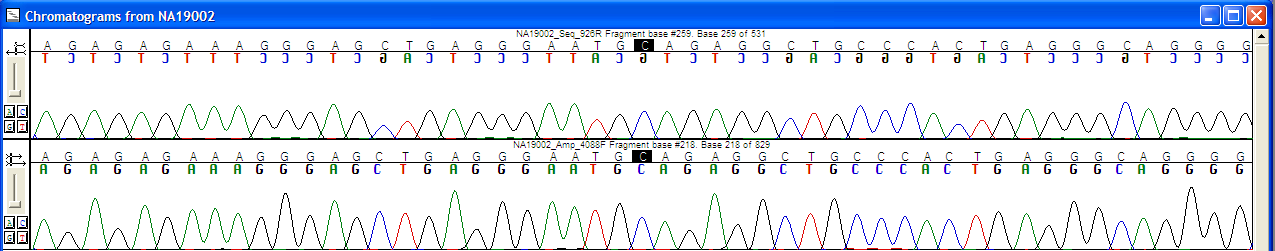


C/G (Sample NA19009)


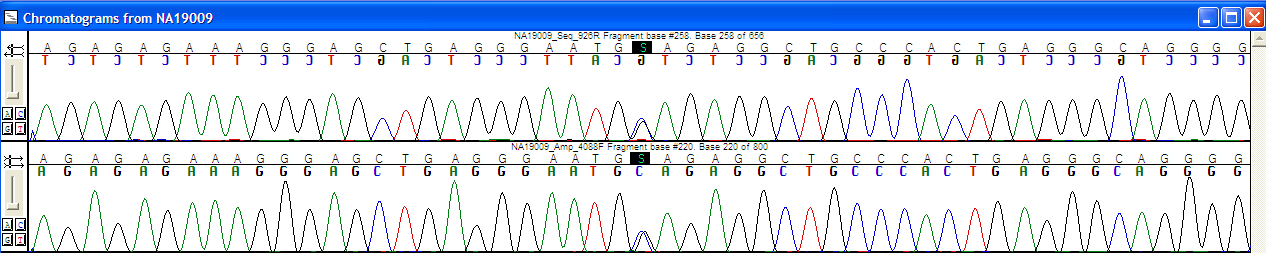


G/G (Sample NA18857)


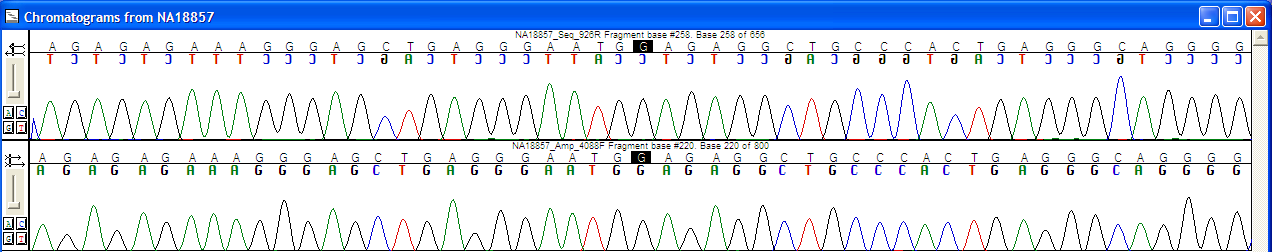


***IL28B* – rs4803219**

C/C (Sample NA19068)


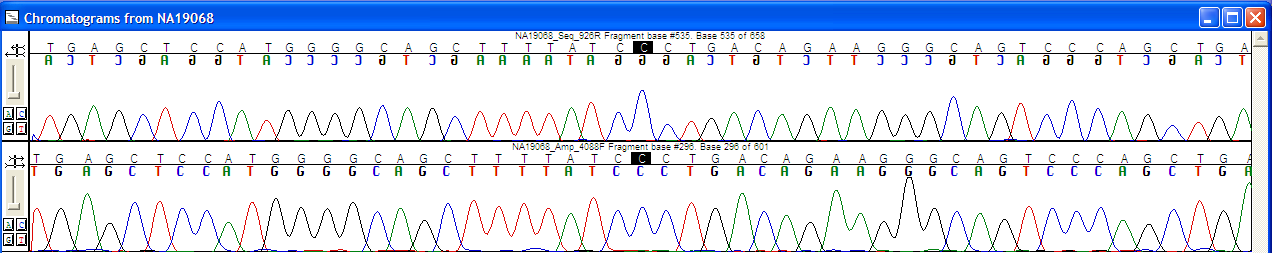


C/T (Sample NA18863)


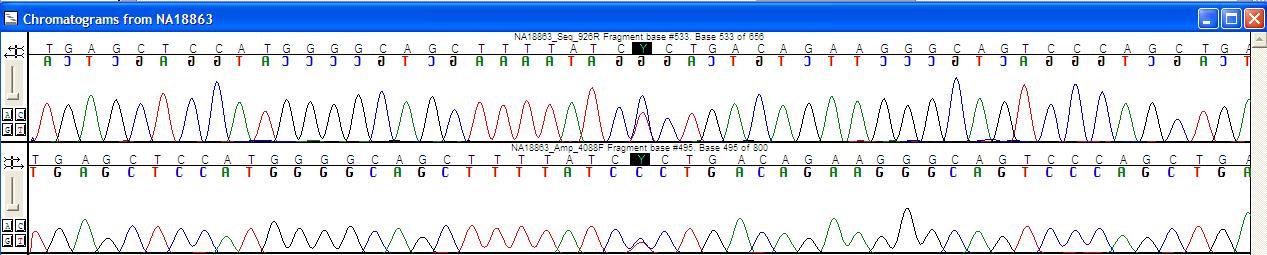


T/T (Sample NA19116)


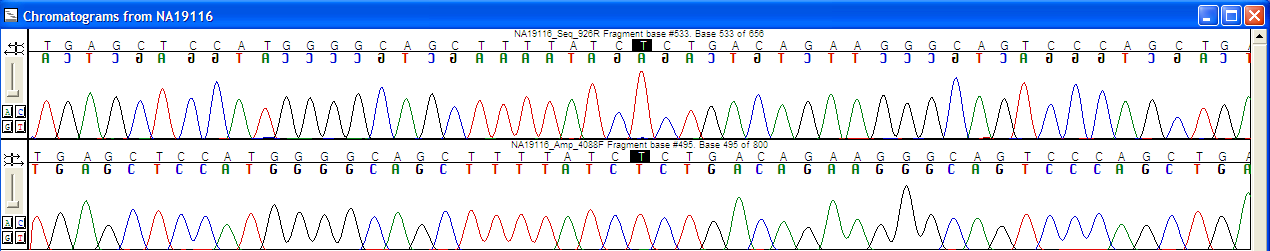


***IL28B* – rs12979860**

T/T (Sample NA17234)


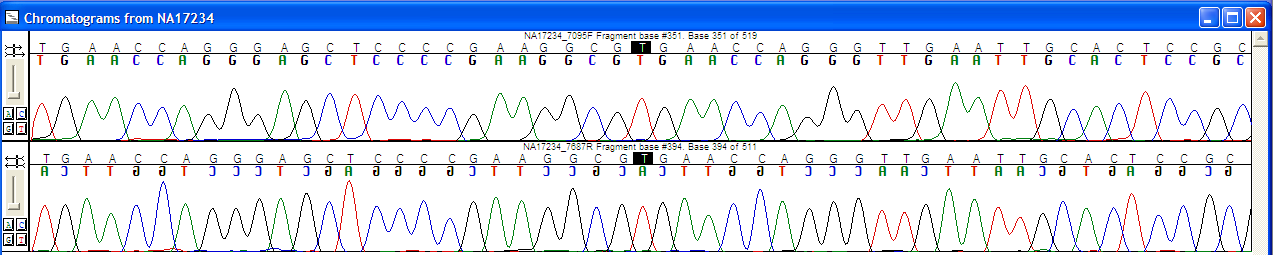


C/T (Sample NA17233)


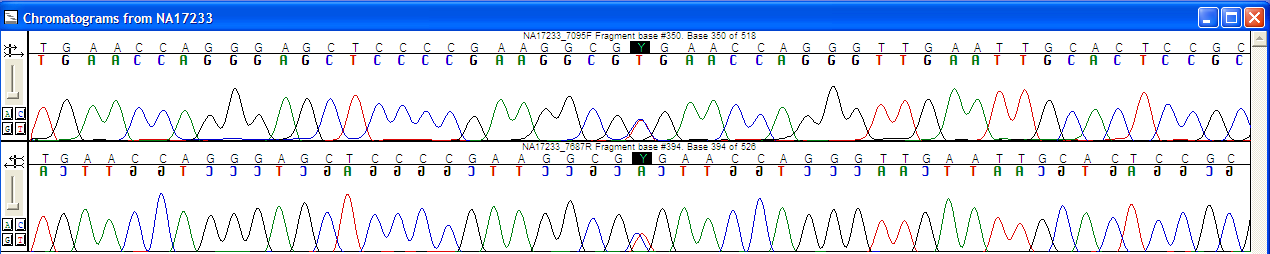


C/C (Sample NA17238)


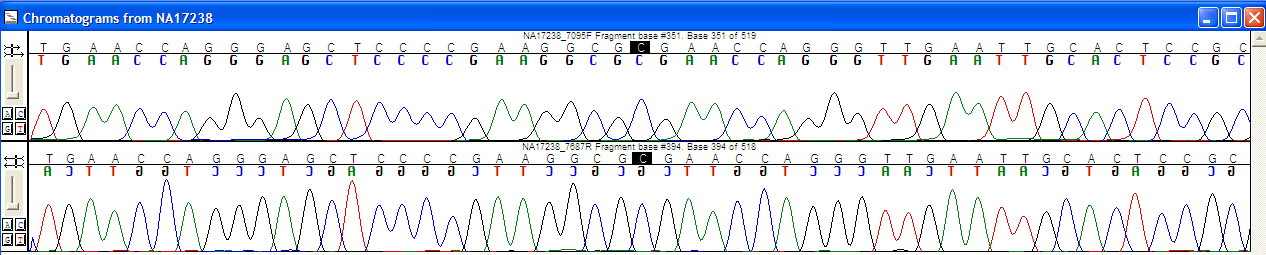


***IL28B* – rs8099917**

T/T (Sample NA17244)


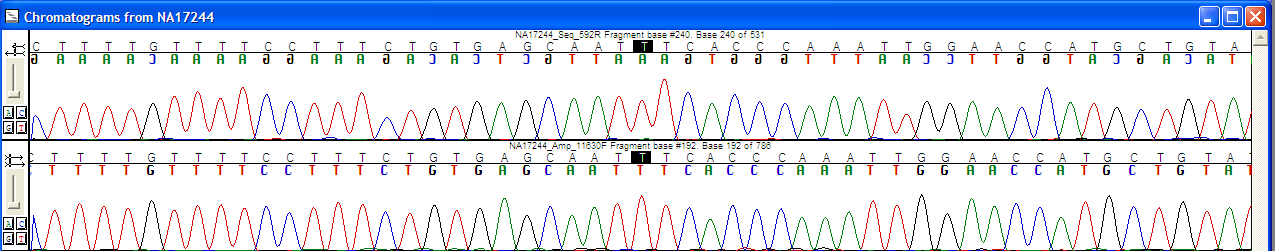


T/G (Sample NA17236)


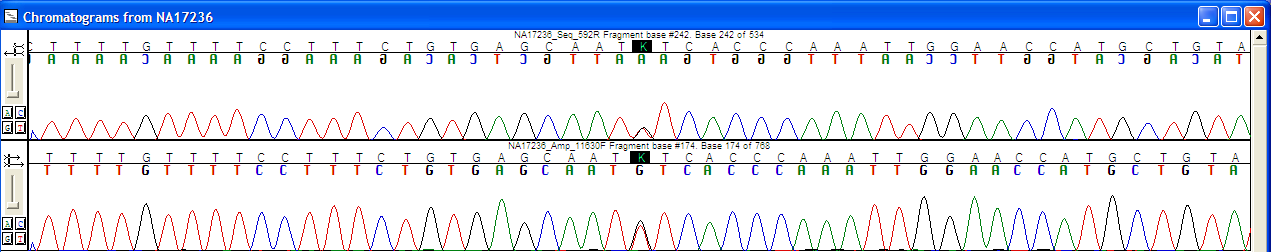


***IL28B* – rs7248668**

A/G (Sample NA17242)


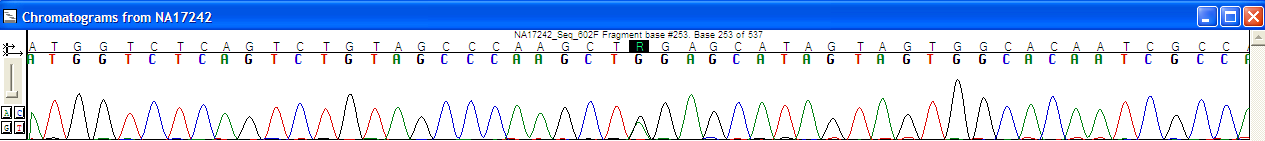


G/G (Sample NA19131)


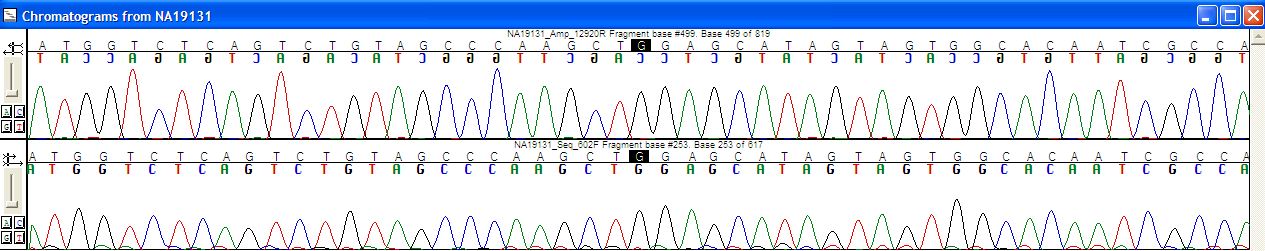


***IL28A* - Reverse complement sequence overlapping rs12979860**

T/T (Sample NA19161)


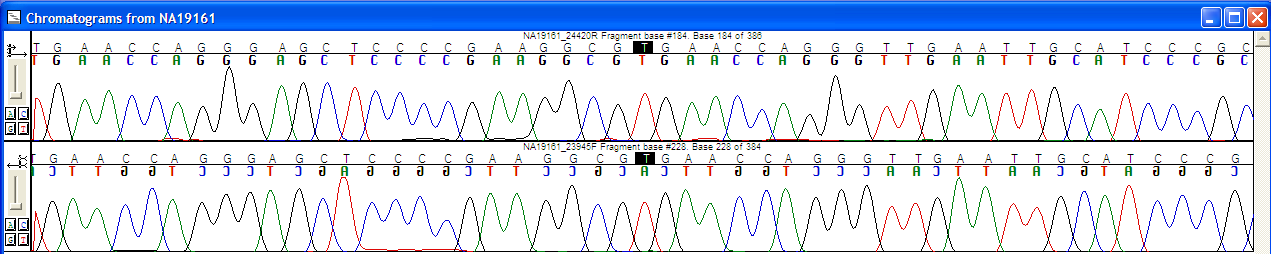


***IL28A* - Reverse complement sequence overlapping rs11881222**

G/G (Sample NA19085)


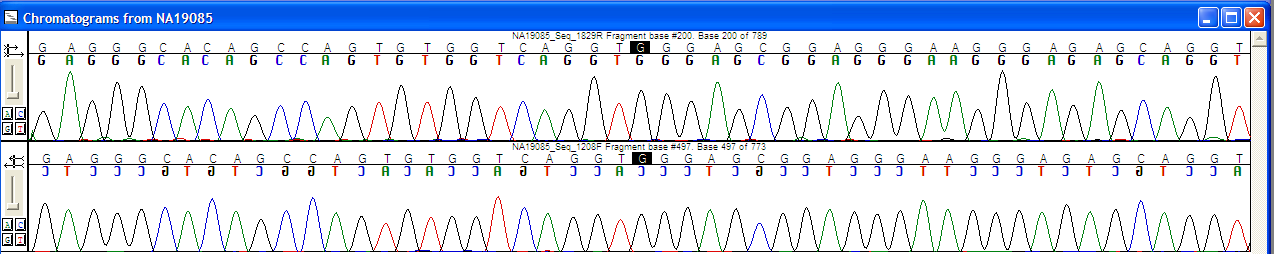


***IL28A* – Reverse complement sequence overlapping rs28416813**

G/G (Sample NA19057)


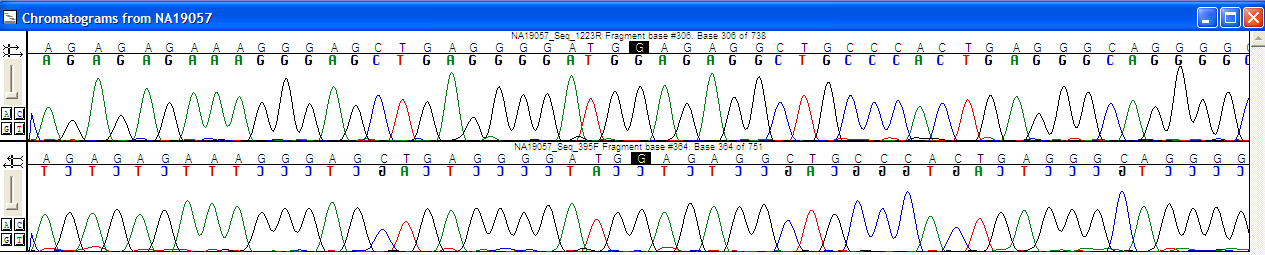


***IL28A* - Reverse complement sequence overlapping rs4803219**

C/C (Sample NA19058)


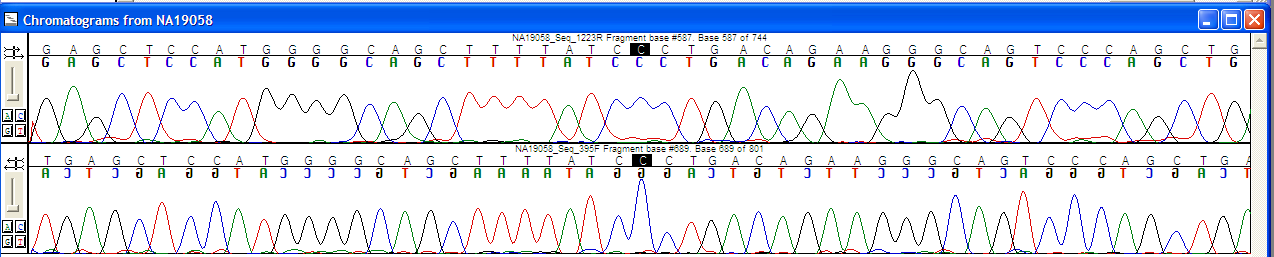


***IL28A* - Reverse complement sequence overlapping rs8103142**

C/C (Sample NA19207)


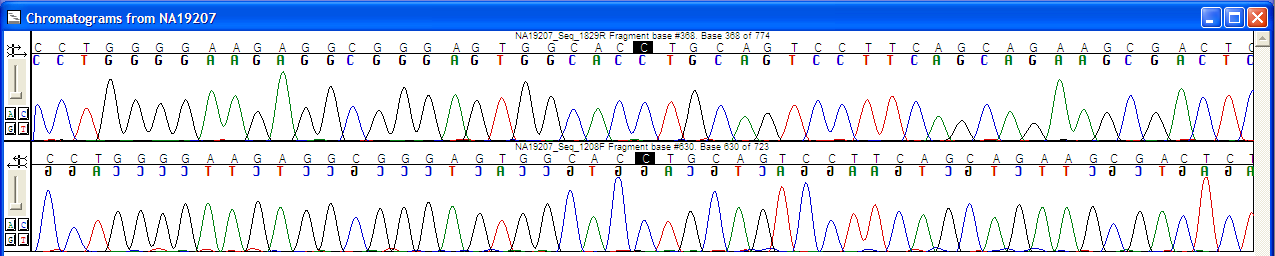


***IL28A* - Reverse complement sequence overlapping rs8105790**

C/C (Sample NA19078)


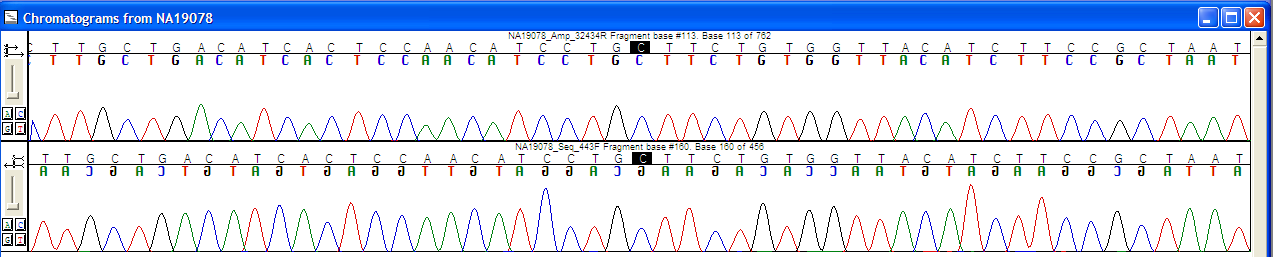

Supplement: Figure S1 — Representative sequencing chromatograms covering the 9 IL28B SNPs as well as the corresponding regions in IL28A . Sanger sequencing chromatograms illustrating the different genotypes found in different Coriell samples for each IL28B SNP and the corresponding regions in IL28A. (DOC) [file pone.0029983.s001.doc]
