# Supplementary material for: Sequence Analysis of the IL28A/IL28B Inverted Gene Duplication That Contains Polymorphisms Associated with Treatment Response in Hepatitis C Patients
Source: PLoS One. 2012 Jan 10;7(1):e29983. doi: 10.1371/journal.pone.0029983 (PMC3254624; doi:10.1371/journal.pone.0029983)
Supplement: Figure S2 — Sequencing chromatograms covering the IL28B rs12979860 SNP for the 48 Coriell Institute DNA sample panel. Sanger sequence chromatograms for the CEU sample panel (NA17233 - NA17297), the JPT sample panel (NA18954 -NA19085) and the YRI sample panel (NA18502 - NA19223). (DOC) [file pone.0029983.s002.doc]

**Figure S2 Sequencing chromatograms covering the *IL28B* rs12979860 SNP for the 48 Coriell Institute DNA sample panel.**

**Figure S14 Sequencing chromatograms covering the *IL28B* rs12979860 SNP for the Coriell Institute Japanese sample panel (NA18954 - NA19001).**
