# Supplementary material for: Sequence Analysis of the IL28A/IL28B Inverted Gene Duplication That Contains Polymorphisms Associated with Treatment Response in Hepatitis C Patients
Source: PLoS One. 2012 Jan 10;7(1):e29983. doi: 10.1371/journal.pone.0029983 (PMC3254624; doi:10.1371/journal.pone.0029983)
Supplement: Table S1 — Primers used to sequence PCR amplicons. Details of sequencing primers used to examine the PCR amplicons encompassing SNP locations in IL28B and the corresponding reverse complement (RC) regions of IL28A. (DOC) [file pone.0029983.s003.doc]

**Table S1. Primers used to sequence PCR amplicons**

| **Target Gene** | **Fragment Size (bp)** | **SNPs covered** | **Amplifying Primers** | **Sequencing Primers** |
| --- | --- | --- | --- | --- |
| ***IL28B*** | 1697 | rs12980275 | 5’-AGCAAGAGGAGGGAAGGAAG-3’ | 5’-AGCAAGAGGAGGGAAGGAAG-3’ |
| rs8105790 | | | 5'-CCCTGGATAAGCCCCTACAG-3' | 5'-CCCTGGATAAGCCCCTACAG-3' |
| 5’-CTGACAGTCAAGATGTCAGC-3’ | | | | |
| 5’-AGTAGCTGAGACTACAGG-3’ | | | | |
| ***IL28B*** | 1115 | rs11881222 | 5'-TGGGTGTCTTTTCCTCATTG-3' | 5'-TGGGTGTCTTTTCCTCATTG-3' |
| rs8103142 | | | 5'-CCTCCAATCCCATCAGAG-3' | 5'-CCTCCAATCCCATCAGAG-3' |
| 5’-AGAGGATATGGTGCAGGG-3’ | | | | |
| ***IL28B*** | 919 | rs28416813 | 5’-TGACCCTTGGAGTGCGGG-3’ | 5’-TGACCCTTGGAGTGCGGG-3’ |
| rs4803219 | | | 5’-ATATGCCAGGAGTGGTGG-3’ | 5’-ATATGCCAGGAGTGGTGG-3’ |
| 5’-CCTTCCTGTAGGTTACCC-3’ | | | | |
| ***IL28B*** | 593 | rs12979860 | 5'-CCAGCAGCTCCAGGATCG-3' | 5'-CCAGCAGCTCCAGGATCG-3' |
| 5'-GCAGGCGCCTCTCCTATG-3' | | | | 5'-GCAGGCGCCTCTCCTATG-3' |
| ***IL28B*** | 1291 | rs8099917 | 5'-TCACCATCCTCCTCTCATCC-3' | 5'-TCACCATCCTCCTCTCATCC-3' |
| rs7248668 | | | 5'-GCACCCAAAGCCTAACCATA-3' | 5'-GCACCCAAAGCCTAACCATA-3' |
| 5’-CAGATGAATTGTGGTGGC-3’ | | | | |
| 5’-AAATCTAAGTGACTCTCTCC-3’ | | | | |
| ***IL28A*** | 476 | RC of rs12979860 | 5'-ACACACCCGTCGCTGACC-3' | 5'-ACACACCCGTCGCTGACC-3' |
| 5'-CTCTCCCGTCCGCTTCTG-3' | | | | 5'-CTCTCCCGTCCGCTTCTG-3' |
| ***IL28A*** | 2500 | RC of rs11881222 | 5’-GCCAATTGGTGAACTGTCAT-3’ | 5’-CCTTCCTGTAGGTTACCC-3’ |
| RC of rs8103142 | | | 5'-CCCAGCTCATCAAGTGTGTCT-3' | 5’-GACTGACTCATGTTTTCC-3’ |
| RC of rs28416813 | | | 5’-AAAACATGAGTCAGTCCC-3’ | |
| RC of rs4803219 | | | 5’-GAGAGGATATGGTGCAGG-3’ | |
| ***IL28A*** | 990 | RC of rs8105790 | 5'-CCACAAATGAGGGGGACA-3' | 5'-CCACAAATGAGGGGGACA-3' |
| 5'-TGGCCAGCTGGTTCTTCTAC-3' | | | | 5'-TGGCCAGCTGGTTCTTCTAC-3' |
| 5’-CCACTGTGACTTAGAAGC-3’ | | | | |
