# Supplementary material for: Sequence Analysis of the IL28A/IL28B Inverted Gene Duplication That Contains Polymorphisms Associated with Treatment Response in Hepatitis C Patients
Source: PLoS One. 2012 Jan 10;7(1):e29983. doi: 10.1371/journal.pone.0029983 (PMC3254624; doi:10.1371/journal.pone.0029983)
Supplement: Table S2 — Novel SNPs identified in IL28A and IL28B gene regions in DNA samples from the Coriell Institute. SNP location and corresponding alleles are indicated using the IUPAC ambiguity codes (R, S, W, Y). (DOCX) [file pone.0029983.s004.docx]

**Table S2. Novel SNPs identified in *IL28A* and *IL28B* gene regions in DNA samples from the Coriell Institute.**

| **DNA Sample ID** | **Gene region** | **SNP location**  **(chr:nucleotide #)** | **Alleles** | **AA change** | **Sequence** |
| --- | --- | --- | --- | --- | --- |
| NA19140 | ***IL28A*** | 19: 39755517 | C/G |  | GCTGCCAGTACCCAT**S**CACGTCCAGGAATCC |
| NA19223 | ***IL28B*** | 19: 39738743 | A/G |  | GTACTGGCAGCGCAC**R**GTCGTGCCTGTCGTG |
| NA18504 | ***IL28B*** | 19: 39738669 | A/G |  | TTATCGCATACGGCT**R**GGCCCCCTCGCCAGG |
| NA19223 | ***IL28B*** | 19: 39738669 | A/G |  | TTATCGCATACGGCT**R**GGCCCCCTCGCCAGG |
| NA18504 | ***IL28B*** | 19: 39738666 | A/G |  | CGCTTATCGCATACG**R**CTAGGCCCCCTCGCC |
| NA19223 | ***IL28B*** | 19: 39738666 | A/G |  | CGCTTATCGCATACG**R**CTAGGCCCCCTCGCC |
| NA18863 | ***IL28B*** | 19: 39738607 | C/G |  | CGGAAGGAGCAGTTG**S**GCTGCCCCCAGCTCA |
| NA17245 | ***IL28B*** | 19: 39736144 | C/T |  | GGTTTTGTTGTTGTT**Y**AAGACAGGGTCTCAC |
| NA19153 | ***IL28B*** | 19: 39735955 | C/G |  | GCTGATGTAGGAAAA**S**TGAAAACACAGCCTC |
| NA19161 | ***IL28B*** | 19: 39735955 | C/G |  | GCTGATGTAGGAAAA**S**TGAAAACACAGCCTC |
| NA19153 | ***IL28B*** | 19: 39735938 | C/T |  | ACAGAAGGGCAGTCC**Y**AGCTGATGTAGGAAA |
| NA19161 | ***IL28B*** | 19: 39735938 | C/T |  | ACAGAAGGGCAGTCC**Y**AGCTGATGTAGGAAA |
| NA19140 | ***IL28B*** | 19: 39735815 | C/T |  | GTTCTTTTCAGTCCC**Y**TCTTCTGGATCTCAG |
| NA18504 | ***IL28B*** | 19: 39735632 | A/G |  | CACAGAGAGAAAGGGA**R**CTGAGGGAATGCAG |
| NA19223 | ***IL28B*** | 19: 39735632 | A/G |  | CACAGAGAGAAAGGGA**R**CTGAGGGAATGCAG |
| NA17241 | ***IL28B*** | 19: 39735548 | A/T |  | TGGCGACAGGAACTGC**W**CCAGTCACGGTCAG |
| NA17241 | ***IL28B*** | 19: 39735517 | A/G |  | CCCCTTGCATCCGGGA**R**AGCCCCGCGGAGCC |
| NA17248 | ***IL28B*** | 19: 39735046 | C/G |  | AGGGGTGGGCCTGACT**S**CCCCTCTCACCTGC |
| NA19116 | ***IL28B*** | 19: 39734773 | C/G | E95Q | AGGGCCAGCTCAGCCT**S**CAAAGCCACGGGGC |
| NA17235 | ***IL28B*** | 19: 39733168 | A/G |  | TCCTGCCAGGGGGAGC**R**CTCACCTTGGGAGG |
| NA18506 | ***IL28B*** | 19:39732601 | A/G |  | GACAACCCAGGATAAC**R**CCCCCATCCCAAAC |
| NA18857 | ***IL28B*** | 19:39732601 | A/G |  | GACAACCCAGGATAAC**R**CCCCCATCCCAAAC |
| NA19131 | ***IL28B*** | 19:39731980 | C/G |  | GATCACTTGAGGTCAG**S**AGTTCGAGACCAAC |
