# Supplementary material for: Sequence Analysis of the IL28A/IL28B Inverted Gene Duplication That Contains Polymorphisms Associated with Treatment Response in Hepatitis C Patients
Source: PLoS One. 2012 Jan 10;7(1):e29983. doi: 10.1371/journal.pone.0029983 (PMC3254624; doi:10.1371/journal.pone.0029983)
Supplement: Table S3 — Genotypes determined for Coriell Institute DNA samples using DNA sequencing, TaqMan® and LabCorp genotyping assays and data extraction from HapMap. Sample NA18502 (bold) is the sample with the discordant result in the LabCorp Inc. assay. n/n indicates the 2 samples that were genotyped in HapMap project, but no results were reported. (DOCX) [file pone.0029983.s005.docx]

**Table S3 Genotypes determined for Coriell Institute DNA samples using DNA sequencing, TaqMan® and LabCorp genotyping assays and data extraction from HapMap.**

| **Sample ID** | **Population** | **Sequencing Genotype** | **TaqMan Genotype** | **HapMap Genotype** | **LabCorp Genotype** |
| --- | --- | --- | --- | --- | --- |
| NA17233 | CEU | C/T | C/T | Not Available | C/T |
| NA17234 | CEU | T/T | T/T | Not Available | T/T |
| NA17235 | CEU | C/T | C/T | Not Available | C/T |
| NA17236 | CEU | T/T | T/T | Not Available | T/T |
| NA17238 | CEU | C/C | C/C | Not Available | C/C |
| NA17239 | CEU | C/T | C/T | Not Available | C/T |
| NA17240 | CEU | C/C | C/C | Not Available | C/C |
| NA17241 | CEU | T/T | T/T | Not Available | T/T |
| NA17242 | CEU | C/T | C/T | Not Available | C/T |
| NA17243 | CEU | C/C | C/C | Not Available | C/C |
| NA17244 | CEU | C/C | C/C | Not Available | C/C |
| NA17245 | CEU | T/T | T/T | Not Available | T/T |
| NA17246 | CEU | C/C | C/C | Not Available | C/C |
| NA17247 | CEU | C/T | C/T | Not Available | C/T |
| NA17248 | CEU | C/T | C/T | Not Available | C/T |
| NA17297 | CEU | C/T | C/T | Not Available | Not Analysed |
| NA18954 | JPT | C/C | C/C | C/C | Not Analysed |
| NA18955 | JPT | C/T | C/T | C/T | Not Analysed |
| NA18979 | JPT | C/C | C/C | C/C | Not Analysed |
| NA19001 | JPT | C/C | C/C | C/C | Not Analysed |
| NA19002 | JPT | C/C | C/C | C/C | Not Analysed |
| NA19009 | JPT | C/T | C/T | C/T | Not Analysed |
| NA19057 | JPT | C/C | C/C | C/C | Not Analysed |
| NA19058 | JPT | C/C | C/C | C/C | Not Analysed |
| NA19063 | JPT | C/C | C/C | C/C | Not Analysed |
| NA19065 | JPT | C/C | C/C | C/C | Not Analysed |
| NA19068 | JPT | C/C | C/C | C/C | Not Analysed |
| NA19075 | JPT | C/C | C/C | C/C | Not Analysed |
| NA19078 | JPT | C/C | C/C | C/C | Not Analysed |
| NA19079 | JPT | C/C | C/C | n/n | Not Analysed |
| NA19084 | JPT | C/T | C/T | C/T | Not Analysed |
| NA19085 | JPT | C/C | C/C | C/C | Not Analysed |
| **NA18502** | **YRI** | **C/T** | **C/T** | **n/n** | **T/T** |
| NA18504 | YRI | T/T | T/T | T/T | T/T |
| NA18506 | YRI | T/T | T/T | T/T | Not Analysed |
| NA18857 | YRI | T/T | T/T | T/T | Not Analysed |
| NA18858 | YRI | C/C | C/C | C/C | C/C |
| NA18863 | YRI | T/T | T/T | T/T | T/T |
| NA18870 | YRI | T/T | T/T | T/T | Not Analysed |
| NA19099 | YRI | T/T | T/T | T/T | Not Analysed |
| NA19116 | YRI | T/T | T/T | T/T | T/T |
| NA19131 | YRI | C/C | C/C | C/C | C/C |
| NA19140 | YRI | C/C | C/C | C/C | C/C |
| NA19153 | YRI | T/T | T/T | T/T | Not Analysed |
| NA19161 | YRI | C/T | C/T | C/T | Not Analysed |
| NA19201 | YRI | T/T | T/T | T/T | Not Analysed |
| NA19207 | YRI | C/C | C/C | C/C | C/C |
| NA19223 | YRI | C/T | C/T | C/T | C/T |
