# Supplementary material for: Sequence Analysis of the IL28A/IL28B Inverted Gene Duplication That Contains Polymorphisms Associated with Treatment Response in Hepatitis C Patients
Source: PLoS One. 2012 Jan 10;7(1):e29983. doi: 10.1371/journal.pone.0029983 (PMC3254624; doi:10.1371/journal.pone.0029983)
Supplement: Table S4 — Concordance of additional genotypes determined for Coriell Institute DNA sample NA18502 compared to HapMap data (Release 24). r2 values from linkage disequilibrium analysis in the YRI population were 0.12 for rs2032586 and rs2235015 on chromosome 6; on chromosome 7 they were 0.06 for rs622342 and rs316019; and on chromosome 19 they were 0.48 for rs8105790 and rs11881222; 0.08 for rs8105790 and rs7248668; and 0.05 for rs11881222 and rs7248668. Analysis also determined that NA18502 was from a female. (DOCX) [file pone.0029983.s006.docx]

**Table S4 Concordance of additional genotypes determined for Coriell Institute DNA sample NA18502 compared to HapMap data (Release 24)**

| \|  \|  \|  \|  \|  \|  \|  \| \| --- \| --- \| --- \| --- \| --- \| --- \| --- \| \|  \| **Chromosome** \| **SNP** \| **Pfizer Lab** \| **HapMap data** \| **Frequency of genotype in YRI** \|  \| \|  \| 1 \| rs1801274 \| CT \| CT \| 50.0% \|  \| \|  \| 6 \| rs622342 \| AA \| AA \| 65.0% \|  \| \|  \| 6 \| rs316019 \| GG \| GG \| 70.0% \|  \| \|  \| 7 \| rs2032583 \| TT \| TT \| 55.0% \|  \| \|  \| 7 \| rs2235015 \| GT \| GT \| 55.0% \|  \| \|  \| 19 \| rs8105790 \| TT \| TT \| 51.0% \|  \| \|  \| 19 \| rs11881222 \| AA \| AA \| 46.7% \|  \| \|  \| 19 \| rs7248668 \| GG \| GG \| 96.6% \|  \| \|  \|  \|  \|  \|  \|  \|  \| |
| --- | --- | --- | --- | --- | --- | --- | --- | --- | --- | --- | --- | --- | --- | --- | --- | --- | --- | --- | --- | --- | --- | --- | --- | --- | --- | --- | --- | --- | --- | --- | --- | --- | --- | --- | --- | --- | --- | --- | --- | --- | --- | --- | --- | --- | --- | --- | --- | --- | --- | --- | --- | --- | --- | --- | --- | --- | --- | --- | --- | --- | --- | --- | --- | --- | --- | --- | --- | --- | --- | --- | --- | --- | --- | --- | --- | --- | --- |
|  |
|  |
